# Systematic Evolution of Ligands by Exponential Enrichment Technologies and Aptamer-Based Applications: Recent Progress and Challenges in Precision Medicine of Infectious Diseases

**DOI:** 10.3389/fbioe.2021.704077

**Published:** 2021-08-10

**Authors:** Yixin Xu, Xin Jiang, Yanhong Zhou, Ming Ma, Minjin Wang, Binwu Ying

**Affiliations:** ^1^ Department of Laboratory Medicine, West China Hospital, Sichuan University, Chengdu, China; ^2^ The First People’s Hospital of Shuangliu District, Chengdu/West China (Airport)Hospital Sichuan University, Chengdu, China

**Keywords:** aptamer, SELEX, infectious disease, precision medicine, biosensor

## Abstract

Infectious diseases are considered as a pressing challenge to global public health. Accurate and rapid diagnostics tools for early recognition of the pathogen, as well as individualized precision therapy are essential for controlling the spread of infectious diseases. Aptamers, which were screened by systematic evolution of ligands by exponential enrichment (SELEX), can bind to targets with high affinity and specificity so that have exciting potential in both diagnosis and treatment of infectious diseases. In this review, we provide a comprehensive overview of the latest development of SELEX technology and focus on the applications of aptamer-based technologies in infectious diseases, such as targeted drug-delivery, treatments and biosensors for diagnosing. The challenges and the future development in this field of clinical application will also be discussed.

## Introduction

Infectious diseases which result from pathogenic microorganisms become one of the most important illnesses in the world (Mosing et al., 2005; Giri et al., 2019). Because of the characteristics of contagion and epidemic, infectious diseases not only endanger public health but also pose serious threats and huge losses to social stability and economic development. Over the past decades, the sudden public health crises including Ebola hemorrhagic fevers, avian influenza, severe acute respiratory syndrome (SARS) or Middle East respiratory syndrome (MERS), as well as COVID-19, have swept out the world and caused a significant impact on society inevitably (Del Rio et al., 2014; Liu et al., 2017; Lycett et al., 2020; Wiersinga et al., 2020; Perra, 2021). Existing pathogen detection methods are difficult to achieve a balance between timeliness, accuracy and cost to meet the requirements of large-scale population screening. In addition, the existence of antibiotic-resistant microbes such as multidrug-resistant *tuberculosis* (MDR-TB), extensively drug-resistant *tuberculosis* (XDR-TB) and methicillin-and aminoglycoside-resistant *Staphylococcus aureus* (MARSA) brings greater challenges to the prevention and treatment of infectious diseases (Fauci et al., 2005; Tan Z. M. et al., 2020; Nang et al., 2021). Therefore, there is an urgent demand to develop rapid, economic, and sensitive early diagnostic assays for pathogens, and also adequate therapeutics of precision medicine for infectious diseases.

Aptamers, also known as “chemical antibodies”, are a class of single-stranded DNAs or RNAs which can target various ligands through non-covalent bonds. Those aptamers are synthetic screened *in vitro* by a selection procedure, commonly known as Systematic Evolution of Ligands by Exponential Enrichment (SELEX). DNA aptamers are more stable and widely used, while RNA aptamers are more likely to form complex structures such as stem, loop, hairpin, G-quadruplex and so on (Breaker, 1997; Lin and Patel, 1997; Wu et al., 2017). These folding 3D structures can increase sequence space coverage and improve space representation, which are beneficial for aptamer-target recognition, thus improving the specificity and affinity of the screened aptamers (Kinghorn et al., 2017). Aptamers have attracted considerable attention because of their exceptional merits such as low synthesis cost, easy chemical modification, high chemical stability and binding affinity, low immunogenicity, good repeatability and reusability (Hong and Sooter, 2015; Yu et al., 2021). To date, thousands of aptamers have been identified, which can be used to identify different targets with high affinity and specificity, such as small metal ions, organic molecules, amino acids, proteins, bacteria, viruses, whole cells and even animals (Cowperthwaite and Ellington, 2008; Zhou and Rossi, 2017). Based on the above advantages, nucleic acid aptamers have been explored as the most promising molecular recognition probes to widely applied in the field of the identification of infectious agents and the therapeutic of infectious diseases.

In this review, the recent advances of SELEX technologies for aptamer selecting of infectious pathogens will be overviewed. Then we will focus on a variety of aptamer-based biosensors for infection detecting and the state-of-the-art aptamer therapeutics and drug delivery systems in the precision treatment of infectious diseases. The current challenges and future prospects of aptamers will also be discussed to provide a direction for the research and development of aptamers.

## Discovery of Specific Aptamers by Systematic Evolution of Ligands by Exponential Enrichment

SELEX was originally developed by Gold and Szostak in 1990 (Ellington and Szostak, 1990; Tuerk and Gold, 1990). Before selecting, an oligonucleotide library consisting of two constant regions at 5′ and 3′ ends and a random region in the middle should be synthesized. The primary library usually contains up to 10^12^–10^15^ different nucleic acid molecules, of which the random region is about 20–40 bp, and the constant regions that flanked are about 20 bp, including restriction endonuclease sites, primer binding sites and RNA promoter binding sites (Zimbres et al., 2013; Hong and Sooter, 2015). Currently, both DNA and RNA libraries are widely used for SELEX due to their distinct advantages.

### General Systematic Evolution of Ligands by Exponential Enrichment Scheme

**FIGURE 1:**
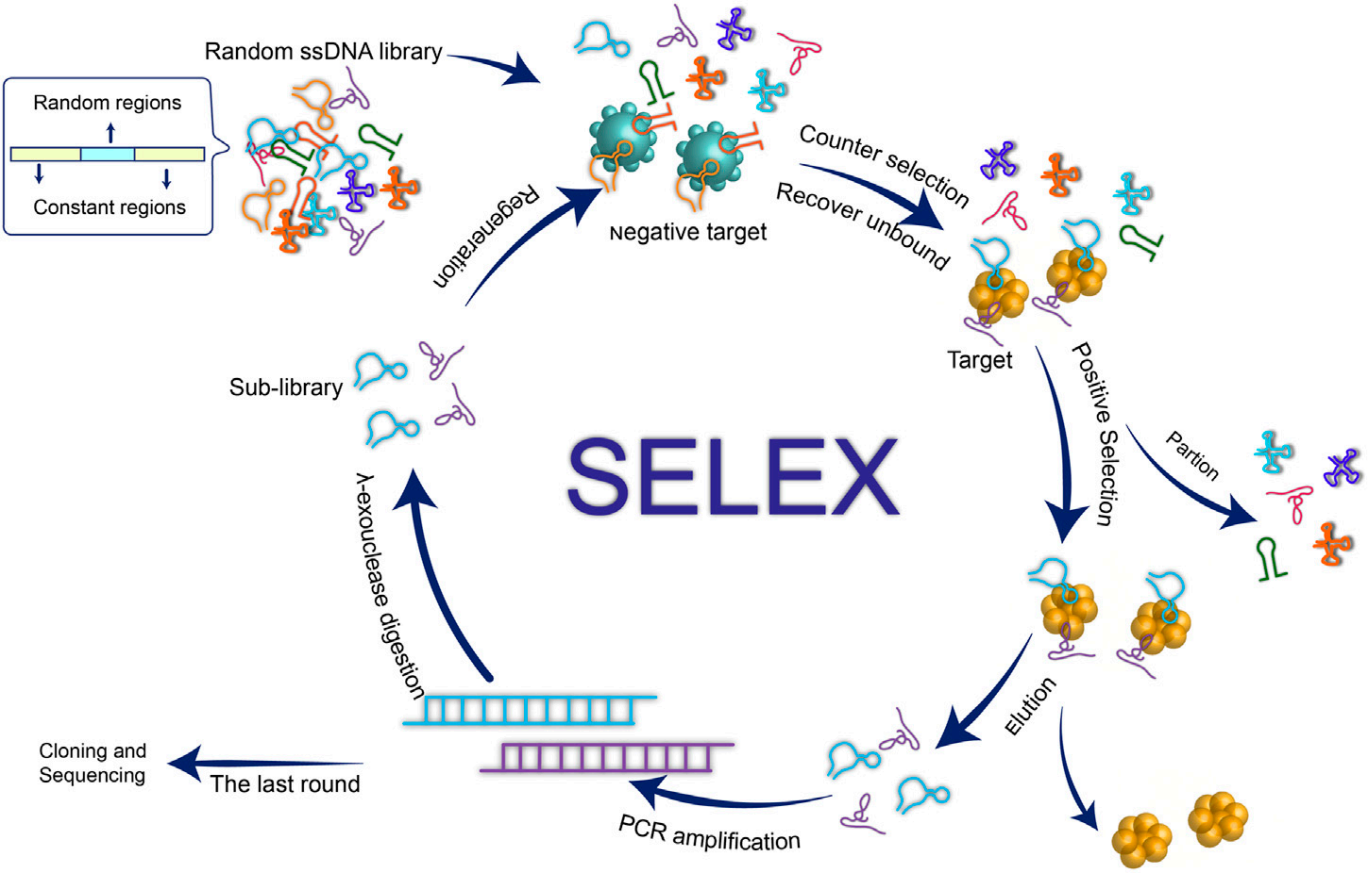
schematic illustration of the aptamer generation by conventional SELEX process.

In brief, a typical SELEX comprises three critical stages: 1) incubate the target molecule with the combinatorial library of nucleic acids *in vitro* to form an aptamer-target complex; 2) partition the complex from the unbound nucleotides and separate the oligonucleotide chain that binds to the target molecule; 3) obtain a sub-library by employing PCR (DNA SELEX) or reverse transcription PCR (RNA SELEX) to amplifying the target-bound sequence (Figure 1). It is worth mentioning that the negative target is usually introduced to improve target specificity by recovering and amplifying the unbound oligonucleotide chain. In this way, the oligonucleotide chain obtained by iterative circles of selecting and PCR amplification is the nucleic acid aptamer of the target. After cloning and sequencing, the identification, binding ability and the secondary/tertiary structure of aptamer can be analyzed subsequently.

### Novel Approaches for Aptamer Selection

Despite the conventional SELEX technology is well-established, the process is tedious and time-consuming, which typically takes up to 20 rounds. At present, various novel SELEX technologies have been developed to improve the shortcomings of the conventional one, further accelerate the speed and efficiency of high-affinity aptamer screening and shorten the selection period. In this respect, the rapid development of SELEX technology provides substantial potential for rapid response to public health emergencies.

The separation of aptamer-bound sequences from the unbound nucleotides is one of the most critical steps in the process of SELEX. Thus, the screening process can be accelerated by optimizing the binding and separation of the target molecule with libraries. Usually, immobilization is involved in binding the targets to a carrier and then incubated with the nucleic acid library (Van Dyke et al., 2016). Common carriers such as magnetic beads, affinity chromatography columns or microfluidic chips. Among them, considerable attention has been given to magnetic beads, as chemical modification of magnetic beads is easy and the magnetic separation method is convenient, fast and effective (McKeague et al., 2010; Hunniger et al., 2014; Xi et al., 2015; Duan et al., 2016; Ma et al., 2018). To date, a variety of aptamers have been successfully selected by magnetic SELEX (Oh et al., 2009; Lai et al., 2014; Wu J.-H. et al., 2018). For example, Hong et al. first proposed a magnetism-controlled chip for Ebola virus aptamers selection by integrating the magnetic bead-based SELEX (Mag-SELEX) with a microfluidic system and by this method they got an aptamer with low dissociation constants and reducing the selecting round to three (Hong et al., 2019). Another novel approach called Capillary Electrophoresis SELEX (CE-SELEX) is based on the difference of electrophoretic mobility between target bounded sequence with the unbound one (Mendonsa and Bowser, 2004a; Mendonsa and Bowser, 2004b). Mosing Renee K et al. successfully obtained aptamers candidate with high affinity to human immunodeficiency virus (HIV) reverse transcriptase by CE-SELEX only after four cycles (Mosing et al., 2005). Although *in vitro* SELEX technology for a single target has been quite mature, especially for proteins, the clinical applications of these aptamers are still limited. The reason is that the properties of a recombinant protein are not the same as those of natural proteins, including conformation, advanced structure and biological activity. Besides, aptamers screened for artificial recombinant proteins may be much less sensitive in identifying natural targets. To overcome this limitation, cell-SELEX, which employs the whole cell as a target, was first proposed in 2003 (Daniels et al., 2003). Recently, a series of aptamers against various pathogens were generated by cell-SELEX, such as *Salmonella typhimurium*, *Escherichia coli*, *Vibrio* parahaemolyticus, Trypanosoma cruzi, and so on (Duan et al., 2012; Nagarkatti et al., 2012; Dwivedi et al., 2013; Kim et al., 2013; Bitaraf et al., 2016; Saad et al., 2020). In addition to the methods mentioned above, many other innovative SELEX strategies have also been developed in the past decades, such as capture-SELEX, *in vivo* SELEX, high-throughput sequencing SELEX and so on. The principles and characteristics of those techniques are summarized in Table 1.

**TABLE 1:** Summary of novel SELEX techniques for aptamer selecting.

Techniques	Description	Advantages	Ref.
Magnetic bead-based SELEX	Immobilize the targets on the magnetic bead, accelerate the process by magnetic separation	Simple and fast operation; short cycle; low cost	Lou et al. (2009), Oh et al. (2009), Oh et al. (2011), Zhou et al. (2013b), Hunniger et al. (2014), Lai et al. (2014), Lai and Hong (2014), Lin et al. (2015), Ansari et al. (2017), Han et al. (2017), Paniel et al. (2017), Wu et al. (2018a), Hong et al. (2019), Leblebici et al. (2019)
Capillary Electrophoresis SELEX	According to the difference of electrophoretic mobility between target bounded sequence with the unbound one	Quick; economic; high efficiency; easy operation without washing procedures	Mendonsa and Bowser (2004b), Mosing et al. (2005), Mosing and Bowser (2009), Cella et al. (2010), Ruff et al. (2012), Jing and Bowser (2013), Yang and Bowser (2013), Dong et al. (2015), Zhu et al. (2019a), Zhu et al. (2019b)
Cell-SELEX	Employ the whole cell as targets	Remain the conformation and bioactivity of protein; obtain aptamers without knowing the molecular target on the cell surface; no need to purify protein before selection; explore new surface protein and biomarkers	Duan et al. (2012), Nagarkatti et al. (2012), Dwivedi et al. (2013), Kim et al. (2013), Bitaraf et al. (2016), He et al. (2019), Song et al. (2019), Gao et al. (2020), Saad et al. (2020), Lin et al. (2021)
Capture-SELEX	Immobilize the oligonucleotides on the solid substrate to further capture the targets	Simple operation of target immobilization; retain the natural structure of the target; especially suitable for small molecular targets	Stoltenburg et al. (2012), Duan et al. (2013), Spiga et al. (2015), Duan et al. (2017), Paniel et al. (2017), Lauridsen et al. (2018), Boussebayle et al. (2019a), Boussebayle et al. (2019b)
*In vivo* SELEX	Use living animal models as selection targets or conditions to generate aptamers	Aptamers were selected in a whole organism ideally; it holds promise for drug delivery and treatment *in vivo*	van Bel et al. (2014), Urak et al. (2016), Zhuo et al. (2017), Wang et al. (2018), Chen et al. (2019), Sola et al. (2020)
High-throughput sequencing SELEX	Conventional SELEX in conjunction with high-throughput sequencing system, sequencing across all the selection round instead of the last one	predominant efficiency and applicability	Roulet et al. (2002), Dittmar et al. (2012), Reiss et al. (2012), Ditzler et al. (2013), Dao et al. (2016), Ruan et al. (2017), Nitta et al. (2019), Asif and Orenstein (2020), Fan et al. (2020), Ishida et al. (2020)

SELEX technology determines that the aptamer target molecules have a very wide range which covers a huge range from metal ions, compounds, peptides, nucleic acids, proteins to cells, and even includes some complex targets, such as viruses, bacteria and other pathogens. Based on SELEX technology, aptamers obtained from specific substances of the pathogens such as surface proteins or key enzymes in physiological processes, are expected to be used for pathogen diagnosis or infection treatment.

## Application of Aptamers Biosensor in Infectious Diseases Diagnosis

Most infectious diseases progress rapidly, and a clear diagnosis is key for effective treatment. Also, efficient pathogen detection methods help control the spread of infectious diseases. Therefore, early, accurate and rapid pathogen diagnosis is of great significance. Currently, the routine diagnostics for infectious pathogen are bacterial culture, polymerase chain reaction (PCR) and immunological detection (Lampel et al., 2000; Byrne et al., 2009; Arvanitis et al., 2014; Rohr et al., 2016; Balmaseda et al., 2017). These approaches are relatively mature, but they inevitably have limitations such as time-consuming, high cost, and tedious operation. Alternatively, although antibody is indispensable in most routine tests, it inevitably meets many limitations, such as laborious and expensive production and identification, batch-to-batch variation, and its biological activity is susceptible to the environment such as pH and temperature (Jayasena, 1999; Nimjee et al., 2005; Rosenbaum et al., 2012; Yu et al., 2021). Aptamers have a very wide range of target molecules, which can be designed for early disease markers, creating conditions for the early detection of pathogens. At the same time, aptamers can be rapidly chemically synthesized in batches with a long shelf-life for storage at room temperature. The comparison of main characteristics between antibodies and aptamers is shown in Table 2.

**TABLE 2:** The characteristics comparison of nucleic acid aptamers and antibodies (Jayasena, 1999; Zhou and Rossi, 2017; Zhuo et al., 2017; Yu et al., 2021).

Features	Antibodies	Aptamers
Substance	polymer peptide	nucleic acid
Specificity	high	high
Affinity	high	high
Immunogenicity	high	no humoral immunity
Production cost	high	low
Stability	unstable	stable
Potential target	limited to immunogenic molecules	no limitation
Development time	6–18 weeks	2–6 weeks
Modification	limited	convenient
Batch-to-batch variation	high	low

Due to those advantages, abundant research has integrated aptamers into biosensors as molecular recognition elements over the past decades. The biological signals received by aptamers can be transformed into the optical signal or electrical signal by the signal converter, then the output signal will be amplified by the electronic system and further be used for the detection of pathogenic microorganisms qualitatively or quantitatively. According to the pathogenic targets, we category these aptamer-based biosensors into three classes: bacteria, viruses and others.

### Detection of Bacteria

The current diagnostic gold standard of bacterial identification is still bacterial culture, subsequent biochemical identification and serological typing (Rauch and Nauen, 2003; Andini et al., 2018; Takeuchi et al., 2018). However, they are time-consuming, usually several days, and have some limitations in the identification of some certain species. In addition, it is less convenient for point-of-care detection. To meet this need, aptamer-based biosensors were widely developed. As an example, an electrochemical sensor for the detection of *Mycobacterium tuberculosis* reference strain H37Rv ATCC 27294 using aptamer technology was introduced by Zhang X. et al. (2019). In this method, H37Rv aptamers layer modified on the bare Au interdigital electrode are recognition probe and oligonucleotides modified with gold nanoparticles (AuNPs-DNA) are signal probe. When H37Rv bacteria was present, it competitively bounds to aptamers, and the displacement of AuNPs-DNA dramatically changing the conductivity. The detection limit of this method is 100 CFU/ml, and it can be used for rapid detection of H37Rv only in 2 h. In one of recent studies, electrochemical aptasensor was devloped for the detection of *Escherichia coli* O157:H7 (*E. coli*) (Qaanei et al., 2021). In this system, the aptamer is employed to improve the selectivity while a reduced graphene oxide–poly (vinyl alcohol) and gold nanoparticles nanocomposite (AuNPs/rGO–PVA/GCE) is used to raise the sensor sensitivity. Consequently, this aptasensor is able to detect *E. coli* as low as 9.34 CFU/ml, with an excellent specificity.

### Detection of Viruses

Many infectious diseases are caused by viral infection, such as acquired immunodeficient syndrome (AIDS), influenza and COVID-19, which has dealt a heavy blow to the world in 2020. These pathogens widely distribute in open systems and are endanger human health and the public environment. For example, the current COVID-19 pandemic in more than 100 countries around the world has infected an untold number of people and caused large numbers of deaths. Thus, it is urgently desirable for cost-effective, rapid and reliable diagnostic methods. Woo et al. designed an aptamer-based fluorescent sensor to detect SARS-CoV-2 RNA in human nasopharyngeal samples (Woo et al., 2020). They intelligently designed a one-pot, ligation-dependent isothermal reaction cascade that consists of a ligation reaction by SplintR ligase and subsequent transcription by T7 RNA polymerase. When target RNA existing, the RNA aptamers of the isothermal reaction products bind to fluorescent dyes and produce a significant fluorescence signal. The detection limit is 0.1 aM. Interestingly, only by redesigning the hybridization regions of the probes, a series of viruses including influenza viruses, MERS and SARS can be detected by this method. Another recent approach was proposed by Liu et al. (2020), which designed a qPCR amplification reaction triggered by two aptamers probes for ultrasensitive detection of serum COVID-19-associated antigens. This method exhibits excellent detection performance and can be conducted within 2 h. In another study by Babamiri et al. (2018), aptamer against HIV-1 was used for the development of an electrochemiluminescence (ECL) sensor. This aptamer-based biosensor showed excellent sensitivity and specificity, with a detection limit as low as 0.3 fM, and can be successfully applied to clinical serum samples analysis.

More importantly, some viruses have multiple subtypes and mutant quickly, which are highly infectious and transmissible pathogens (Shim, 2011; Li et al., 2019; Ma and Ma, 2020). Therefore, the detection of mutation is becoming a top priority in the field of methodological research. At present, molecular methods such as PCR and DNA sequencing are powerful tools to obtain information on mutant status, but these methods could not satisfy the expectation for extensive disease screening because of the need for special equipment and expensive consumables (Escadafal et al., 2014; Ma et al., 2015). Recently, Wang et al. established a highly sensitive platform for detecting SARS-CoV-2 and its mutated variants based on a CRISPR-Cas13 transcription amplification principle (Wang et al., 2021). They employed light-up RNA aptamers as the sensitive output of amplification signals, achieving sensing of as low as 82 copies of SARS-CoV-2. Moreover, this platform was applied to strictly identify the key mutation of the SARS-CoV-2 variant, D614G, which increases viral stability and flexibility and further enhances replication and transmission.

### Detection of Other Pathogens

Aptamer-based biosensors are used to detect several other pathogens alternatively. Protozoan parasite infection remains one of the major public health problems in some underdeveloped and developing countries with poor sanitation and economic backwardness. Thus, the application of detections that require expensive equipment or complex laboratory sites is significantly limited in these areas. In this case, a deal of aptamer-based biosensors has been developed for the identification of parasites due to their low cost, simplicity, portability (Lee et al., 2012; Singh et al., 2019a; Singh et al., 2019b; Frezza et al., 2020; Minopoli et al., 2020). Take the diagnosis of malaria as an example, Singh et al. established instrument-based and instrument-free approaches for pan malaria and P. falciparum species based on aptamers specific to Plasmodium lactate dehydrogenase (PLDH) and Plasmodium falciparum glutamate dehydrogenase (P*f*GDH) respectively (Singh et al., 2019a). They successfully overcame the false-negative limitation of traditional microscopic examination of Giemsa-stained thick blood films and achieved an ultrasensitive detection with a low cost (∼0.10 $ per test) (Mikhail et al., 2011; Thongdee et al., 2014; Bin Dajem, 2015; Sumari et al., 2016; Habyarimana and Ramroop, 2020). In addition, Fu developed an indirect blocking enzyme linked aptamer assay (ib-ELAA) for the detection of *Mycoplasma gallisepticum* (*M. gallisepticum*), which was the major pathogen of chronic respiratory disease (Fu et al., 2021b; Fu et al., 2021a). In this method, they initially screened out the aptamer Apt-236 which can bind to PvpA protein of *M. gallisepticum* with high affinity, and further integrated Apt-236 into ib-ELAA and successfully applied in the detection of clinical chicken sera sample. Similarly, a great many of aptamer-based methods for the detection of pathogenic parasites (Homann et al., 2006; Bruno et al., 2014; Ospina-Villa et al., 2018; Ospina, 2020), *mycoplasma* (Fu et al., 2014; Liu Y. et al., 2019; Wan et al., 2020), several fungal species (McKeague et al., 2010; Barthelmebs et al., 2011; Ma et al., 2014; Wu S. et al., 2018; Liu M. et al., 2019; Han et al., 2021) have also been developed.

The comparison of representative aptasensor performance in the detection of the various pathogen is summarized in Table 3.

**TABLE 3:** Comparison of aptasensor performance in the detection of various pathogen.

Type of pathogen	Target	Aptamer	Detection method	Lod	Linear range	Detection time	Specificity	Ref.
*Bacteria*	*Mycobacterium tuberculosis*	H37Rv aptamers	Electrochemical	100 CFU/ml	1×10^2^–1 × 10^7^ CFU/ml	2 h	90%	Zhang et al. (2019a)
	*Staphylococcus aureus*	*S.aureus* aptamer	Fluorescent	39 CFU/ml	80–8 × 10^6^ CFU/ml	NR	high	Cai et al. (2019)
	*L. monocytogenes*	LM6-116	Fluorescent	10 CFU/ml	10–1 × 10^6^ CFU/ml	NR	high	Guo et al. (2020)
	*Escherichia coli*	E1	Fluorescenct	3.7 × 10^2^ CFU/ml	6×10^3^–3.75 × 10^6^ CFU/ml	135 s	high	Zhang et al. (2019b)
Viruses	SARS-CoV-2	D614G variants aptamer	Fluorescent	82 copies	100–1,000 copies	20 min	high	Wang et al. (2021)
	HIV-1	HIV aptamer	ECL	0.3 fM	3.3 fM–0.3 nM	NR	high	Babamiri et al. (2018)
	HBV	HBsAg aptamer	Chemiluminescent	0.05 ng/ml	1–225 ng/ml	NR	high	Xi et al. (2018)
	Influenza	Influenza nucleoprotein aptamer	lateral flow immunoassays	0.26 pg/ml	0.01–10 ng/ml	10 min	high	Kang et al. (2019)
	Norovirus	Aptamer-6-FAM, Bt-Apt-Fc	Microfluidic	100 pM	100 pM - 3.5 nM	35 min	high	Chand and Neethirajan, (2017)
*Other pathogens*	*P. falciparum*	PLDH/P*f*GDH aptamers	Colorimetric	0.55 pM/1.34 pM	1 pM - 100 nM	35 min	high	Singh et al. (2019a)
	Trypanosoma cruzi	Apt68	PCR	0.33 parasites/ml	NR	NR	high	Dunning et al. (2014b)
	Leishmania	Leishmania aptamer	Fluorescent	∼100 ng/2 ml sample	0–1,000 ng	∼1 h	NR	Bruno et al. (2014)

NR, not report.

## Application of Aptamers in Infectious Diseases Treatment

At present, the therapeutic of infectious diseases is mainly based on the principle of symptomatic treatment or specific anti-pathogen treatment. However, antimicrobial resistance, high viral genomes mutation variability and escaping the host immune response make most medications and vaccines inefficient (Finlay and McFadden, 2006; Labella and Merel, 2013; Dunning et al., 2014a; Fall-Malick et al., 2014; Ferir et al., 2014; Lazarevic, 2014; Marascio et al., 2014; Sahu, 2015; Wandtke et al., 2015). It is worth mentioning that the effect of antiviral therapy is not ideal for all patients and side effects caused by many existing antiviral drugs may lead to other diseases than primary affection. For instance, the most effective therapy for patients with hepatitis C (interferon alfa-2b plus ribavirin) benefits only about 50% of cases (Manns et al., 2001; Sarhan et al., 2020), whereas such therapeutic regimen usually be associated with numerous adverse effects (Fried, 2002; Nishimura et al., 2002; Fisher et al., 2004; Negro, 2010; Pazienza, 2011; Gull et al., 2019). Many studies confirmed that aptamer, as a promising candidate, can target the key molecules in bacterial physiological processes or viral surface proteins, and treat the infection effectively by inhibiting viruses penetrating the cells, disrupting the activity of enzymes related to viral replication or regulating immune response (Bellecave et al., 2008; Gopinath et al., 2012; Hwang et al., 2012; Torabi et al., 2020).

### Aptamer-Based Therapeutics

Precision medicine holds great promise to harness the benefits of aptamers that can bound to targets with high specificity and affinity for targeted treatment of a variety of diseases. Such therapeutic aptamers function mainly in the following two ways: 1) aptamers function as antagonists to disrupt the function of a pathologic target protein and block the interaction of disease-associated targets by specifically binding to target; 2) aptamers function as agonists to increase the ability of the target receptors. For instance, Lee et al. reported an RNA aptamer against the Hepatitis C virus (HCV) nonstructural protein 5B can effectively inhibit HCV replication and suppressed HCV infectious virus particle formation (Lee et al., 2015). HIV integrase is considered necessary for retroviral replication, which is a primary target for the therapy of AIDS (Shoji et al., 2002). Thus, aptamers as potential anti-HIV integrase inhibitors have drawn much attention from researchers. Pang and his colleague designed an anti-HIV lentivirus vector consist of shRNA, ribozyme and RNA decoy (Pang et al., 2018). By screening aptamers against integrase and incorporating these aptamers in shRNA, they successfully observed interference and inhibition to transcription of HIV in cell cultures. Also, many other aptamers against Tat protein, gp120, reverse transcriptase, nucleocapsid protein were developed for further exploit research of antivirus therapy (Mufhandu et al., 2012; Aeksiri et al., 2014; Nguyen et al., 2020; Zhang et al., 2020). COVID-19 has wreaked havoc all over the world, but no specific treatment has been developed yet. Liu and his colleague developed an aptamer that specifically targets the spike protein of the coronavirus SARS-CoV-2, which is the critical role of viral infection (Liu et al., 2021). When the receptor-binding domain (RBD) of the spike protein of the coronavirus SARS-CoV-2 binds to the human angiotensin-converting enzyme 2 (ACE2), an infection cascade is triggered (Schmitz et al., 2021). They proved that this aptamer effectively protects host cells from infection by blocking the interaction between spike protein and ACE2 receptor. This exciting report is fueling hope in the field of COVID-19’s therapy, it also brings up new opportunities for aptamer-based treatment.

Despite aptamer-based therapy shows huge potential, their inherent physicochemical characteristics affect pharmacokinetic properties in some way, which may limit their widespread clinical application. The most critical problems are nuclease degradation and rapid renal filtration. Unmodified nucleic acid aptamers have an average half-life of fewer than 10 min for the susceptibility to nucleases which abundantly exist in biological fluids (Lakhin et al., 2013). To increase its biostability and prolong the *in vivo* half-life, chemical modifications are typically introduced such as replacing 2′-OH with fluoro (F), amino (NH2), or O-methyl (OCH3) groups at the 2′ position (Morita et al., 2018). Since the average diameter of 5–30 kDa aptamers is less than 5nm, which is smaller than the glomerular filtration threshold (i.e., 30–50 kDa), aptamers are inevitably rapidly excreted through renal filtration (Guo, 2010; Morita et al., 2018). To overcome this disadvantage, many macromolecular substances such as proteins, cholesterol, liposomes, high molecular mass PEG or nanomaterials are involved to modify aptamers, and there is indeed a significant improvement in some reports (Fisher et al., 1976; Drolet et al., 2000; Rusconi et al., 2004; Burmeister et al., 2005; Chen et al., 2015; Heo et al., 2016). However, chemical modification is a double-edged sword. Serious allergic responses caused by biomaterial, non-specific immune activation, tissue toxicity caused by drug metabolism and other undesirable side effects have been reported (Geary et al., 2003; Waring, 2010; Wong and Goldberg, 2014; Lincoff et al., 2016; Morita et al., 2016). Thus, it is necessary to cautiously improve and optimize the formulations or administration routines of aptamer therapy.

### Aptamers as Intelligent Chemical Drug-Delivery Systems

The other ingenious anti-infective therapeutic strategy is to employ aptamers as intelligent messengers of therapeutic agents, such as small interfering RNA molecules and ribozymes (Dey et al., 2005; Romero-López et al., 2012; Zhu et al., 2012; Wandtke et al., 2015). In the broad area of drug delivery system, aptamers have also been extensively sought after due to the inherited merits: relatively small physical size, versatile structure, quick chemical production, flexible chemical modification, high stability, and lack of immunogenicity. Through chemical modification and bioconjugation, a variety of therapeutic agents increase their stability and bioactivity without change the primary characteristics (Fattal et al., 2018). The targeting of aptamer increases the local drug concentration, thus improving the therapeutic efficacy whilst reducing the systemic toxic and side effects of the drug. Therefore, many cell-specific aptamers are explored to conjugate with chemical entities including chemotherapeutic agents, siRNA, nanoparticles for targeted delivery of drugs.

For the development of aptamers as drug-delivery systems, one example is anti-gp120 aptamer for the treatment of AIDS (Zhou J. et al., 2013). A viral surface protein, gp120, is closely related to viral infection. HIV-1 infects target cells through binding gp120 to cellular receptor CD4 and chemokine receptors such as CCR5 or CXCR4. In this research, Zhou and his colleague employed an anti-gp120 aptamers as siRNA delivery vehicles, effective delivery viral inhibiting siRNA *in vivo* and potent inhibition of HIV-1 replication. Similarly, *Pan* et al. designed an ingenious system which employed bispecific circular aptamers (bc-apts) to specifically tether protein cargoes and cellular membrane proteins (Pan et al., 2020). This strategy achieved the specific delivery of functional therapeutic proteins, and the deactivation of functional proteins was also avoided. Furthermore, the bioactivity of the drug in the lesion was specifically increased. Yan et al. reviewed the design and application of aptamers as drug-delivery systems in the photodynamic platform of targeted therapy (Yan et al., 2021). In this review, they focused on the application of aptamers-targeted photodynamic therapies which achieve controlled and accurate delivery of therapeutic drugs to the lesion sites and obtained excellent photodynamic therapy efficiency. Another advantage of an aptamer-based targeted delivery system is that it can delay the evolution of resistance and improve the efficiency of the antimicrobials on already resistant pathogens. Ucak et al. used *Staphylococcus* aureus-specific aptamer to functionalize methicillin, which is an antibiotic for serious infectious caused by Gram-positive bacteria (Ucak et al., 2020). By limiting the amount and dosage during therapy, they proved that the novel delivery system was significantly effective in reducing minimum inhibitory concentration (MIC) values. Therefore, the aptamer-based targeted delivery system is a promising method for the treatment of infections caused by antibiotic-resistant bacteria. In addition to the examples above, multiple types of research have shown the extraordinary ability of aptamer in drug target-delivering (Nimjee et al., 2005; Shiang et al., 2013; Hahn, 2017; Chonco et al., 2018; Fattal et al., 2018; Tan X. et al., 2020).

## Conclusion and Future Perspectives

Taking advantage of low cost, easy chemical modification, high specificity and binding affinity, low immunogenicity, aptamers have been used as an alternative to antibodies in the development of aptamer-based technologies in the past decades. The development of biomedical technology has enabled a comprehensive exploration of the screening technologies and practical applications of aptamers. In this review, we comprehensively discussed the recent progress in the development of SELEX technology and aptamer-based applied research in various types of infectious diseases.

Up to now, aptamers were intensively integrated into the biosensor strategies as molecular recognition elements. Compared with conventional diagnosis methods, aptamers-based biosensors strategies had obvious advancement in sensitivity and reliability which could improve diagnostic performance, thus lead to intervention at an earlier stage and avoid the spread of infectious diseases. Furthermore, as a class of single-stranded nucleic acid, aptamers showed outstanding advantages on cost and manufacturability, so that the development of aptamer-based biosensors could be conducive promote the popularization and improvement of infectious diseases diagnosis techniques in community hospitals. Last but not the least, the portability makes biosensors an alternative to traditional methods in point-of-care diagnostics and even more diverse medical settings such as epidemic areas. Aptamers could be also applied in biotherapy and drug-delivery systems. Due to low immunogenicity and high targeting ability, aptamer-based therapy could increase the drug concentration in local lesions, thus improve the therapeutic effect and reduce the toxic and side effects of drugs. Aptamers were also intelligent in solving problems of antimicrobial resistance and viral genomes mutation variability. Therefore, aptamers are expected to be promising in the therapeutic of infectious diseases.

However, there remain several challenges limiting the clinical application of aptamers. Firstly, aptamers for some complex pathogens are still limited because of the limitations of the current SELEX technology. Nevertheless, those problems can be ameliorated by optimizing the critical factors in the SELEX process in near future, such as the concentration of target molecules, the separation method of aptamers, PCR reaction conditions, the number of screening rounds and so on. Besides, the biostability and toxicity of aptamers, as well as the degradation of unmodified aptamers by nuclease in serum and rapid removal by renal filtration remain to be explored. The side effects caused by the metabolism of aptamer are also a problem to be reckoned with. Therefore, significant refinements of biochemical modification and rigorous administration routines of aptamer-based therapy are still needed in future research. Fortunately, in spite of all the challenges mentions above, the transition from aptamer-based basic research to clinical application is taking place, although slowly.

Overall, we foresee a promising prospect for aptamer-based technologies in precision medicine of infectious diseases. Shortly, with many research and development activities going on in this field, we envision that practical and commercial biosensors and novel drugs for clinical diagnosis and precise therapy are very close to realization, and consequently, that will significantly reduce the human diseases and economic burdens.
